# Application of Spatially Distributed Field of Electric Impulses for Correction of Angiogenesis in Ischemic Muscular Tissue

**Published:** 2010-12

**Authors:** V.S. Kublanov, I.G. Danilova, I.F. Goette, I.A. Brykina, M.A. Shalyagin

**Affiliations:** 1 *Ural Federal University, Ekaterinburg, Russia;*; 2 *Institute of Immunology and Physiology of Ural Branch of Russian Academy of Sciences, Ekaterinburg, Russia;*; 3 *Ural State Medical Academy, Ekaterinburg, Russia;*; 4 *Institute of Medical Cell Technology, Ekaterinburg, Russia*

**Keywords:** spatially distributed field of electric impulses, ischemia, angiogenesis, endogenous intoxication

## Abstract

Influence of spatially distributed field of electric impulses in a projection to cervical ganglions of the sympathetic nervous system on angiogenesis in ischemic muscular tissue of a rat’s shin has been studied. It is revealed that blood supply of animals, influenced by the field, is restored through increase in quantity of capillaries in ischemic tissues, and number of products of endogenous intoxication is reduced.

## INTRODUCTION

Steady growth of number of the diseases caused by blood flow disorder in arterial vessels, demands new ways and stimulation methods for angiogenesis in ischemic tissue (2, 3, 8). It is known that the ischemia arising on the basis of vessels space narrowing and blood circulation disorder is accompanied by formation of collateral blood circulation which does not always compensate insufficiency of arterial blood inflow. In this case delivery of oxygen to tissues decreases, hypoxia of tissue is developed. The quantity of underoxidated products, molecules of average and low molecular mass (MAM), including products of cells disintegration and a large amount of factors of inflammation, proinflammatory cytokines grows. Blood viscosity increases, thrombocyte and erythrocyte aggregation amplifies. Thrombocyte aggregates block the microcirculatory channel, intensifying ischemia (7, 8).

At the present time effects of medicamental, surgical and well-known physiotherapeutic methods of treatment of ischemic diseases are concentrated on restoration of blood flow by means of decrease in tone and expansion of vessels or development of collateral blood circulation (4–9, 18).

However, the most perspective are the methods aimed at stimulation of angiogenesis in ischemic tissue (9–13). The basic stimulus to angiogenesis in physiological and pathological conditions is hypoxia. The induced by hypoxia Factor-1 (HIF-1) causes expression of the Vessels Endothelium Growth Factor (VEGF) and its receptors. VEGF stimulates migration of monocytes and endotelial cells, formation of proteases and NO with endotelial cells and increase of vascular permeability that promotes «transudation» of growth factors of the albuminous nature and migration of cells in perivascular space (1). The major stimulator of stabilization of primary high-permeability vascular structures and angiogenesis is the increase in pressure of shift above the place of occlusion, caused by blood-groove increase that promotes an expression of adhesive molecules of endothelial cells and the subsequent accumulation in a vessel wall of monocytes, secreting a considerable quantity of growth factors, including the growth factor of fibroblasts (FGF) and VEGF (1).

Methods of angiogenic therapy include: influence by exogenous growth factors in the form of recombinant fibers, genetic designs or stem cells, mobilization of endogenous stem cells from a marrow or tissue depot, and also a combination of these influences. However recombinant fibers have the short period of a semi life in a blood flow, and reception of stem cells is an expensive and labor-consuming method with possible complications in the form of an unpredictable differentiation of stem cells or formation of tumors (9, 11–13).

It is known that circulation control is provided with interaction of local humoral mechanisms and vegetative nervous system which by means of regulation of a tonus of a vascular wall defines a blood flow parity in organs and tissues with level of their functional activity (17). The compelled insufficient functional load with impassability of the main arteries of legs owing to painful sensations and ischemic condition of the muscular tissue, taking place at the given pathology, leads to disturbance of vegetative regulation of a tonus of vessels. The quite perspective method to correct a condition of vegetative nervous system is the method of an electrical stimulation of sympathetic ganglions by the focused rotating spatially distributed field of impulses of a current (FRF). This field is formed by apparatus like «SIMPATOKOR» (14). The central part in treatment of various diseases, with application of these apparatus, is the dynamic correction of activity of sympathetic department of vegetative nervous system which consists of alternating stages of influence by a field in a projection of cervical ganglions of sympathetic department and pauses between influences (15). Influence on cervical ganglions by the field of impulses of a current should cause augmentation of a blood flow above the area of occlusion and stimulate artery genesis in the ischemic limb (1), since superfluous excitation or inhibition of sympathetic knots, including cervical ganglions, can be filtered to underlaying sympathetic ganglions.

The purpose of this work was to study the possibility of stimulation of an angiogenesis in ischemic limbs of rats through a dynamic correction of activity of sympathetic nervous system by the focused spatially distributed field of impulses of current.

## MATERIALS AND METHODS

### The characteristics of laboratory animals and their separation into experimental groups

The research was carried out on not purebred rats-males weighing 200–250 gr according to the advice of the international committee on the humane treatment of laboratory animals. For the control and experiments healthy animals of one age were used. Rats were held in conditions of a usual laboratory vivarium with natural change of day and night. In the tests 20 animals divided into 4 groups were used. The first group was formed with intact healthy animals. The second group consisted of animals immobilized (fixed on the table) for 15 minutes a day, during 5 days, which was necessary for the application of the apparatus “Simpatocor”. In the third group of animals we modeled ischemia of a muscle of a back paw shin. The fourth group consisted of rats undergone similar operation and in 12 days (the period necessary for development of ischemia) their cervical ganglions of sympathetic nervous system were influenced with the focused spatially distributed field of impulses of current during 5 days, 1 procedure a day.

### The correction technique of activity of the sympathetic nervous system by “Simpatocor” with ischemia of a back limb shin of a rat

To carry out the procedure the animals were temporarily immobilized on a specially equipped laboratory table. On the areas in the projection of the left and right cervical ganglions of a sympathetic nervous system, daily for 15 minutes within 5 days, multielement electrodes of “Simpatocor” intended for an electrical stimulation of laboratory rats were established.

To provide the equivalence of functional processes in a human body and a laboratory rat's body the criteria of static and dynamic similarity were used, which have allowed to formulate requirements to biotropic characteristics of field of electric impulses in experiment. Proceeding from it diameter of electrodes of device “Simpatocor-M” applied in experiment, is chosen equal d_mod_=0,1 mm, and frequency of field of electric impulses is chosen equal 300 Hz (14).

### The technique of a back limb ischemia

Under an etherization on a femoral artery of the animals 2 ligatures were imposed at 5 mm from each other, then the vessel was accurately cut and the wound was sutured up level-by-level. The choice of the research period (12 days after operation) corresponds to the period of development of ischemic disturbances in a rat's shin.

### The technique of histological and immunohistochemical research of a muscular tissue

For histological research a sample of muscular tissue from the ischemic area was taken. To analyze the morphological picture (the presence of an edema, the state of the retractor apparatus and the kernels of muscular fibers) and to reveal the capillary bed, the sections were coloured histochemically on an alkaline phosphatase according to the method of Gomori. For the purpose of revealing a capillary bed and calculation of quantity of capillaries, paraffinic sections were coloured also immunohistochemically on CD31 (Mouse Anti-Rat CD31, clone: TLD-3A12) – a specific marker of endotheliocytes. For the immunohistochemical staining the highly specific monoclonal antibodies are applied, capable to be bound only with one antigen with the formation of a steady complex “antigen-antibody”. This approach allows to identify very precisely the cells interesting for a researcher according to their marker antigens (for the endotheliocytes forming the wall of capillaries, it is CD31). Calculation of the quantity of capillaries in a muscle was carried out in 20 casual areas 0,017 mm^2^ each with the subsequent recalculation on 1 mm^2^.

### The technique of biochemical research of the samples of blood

The damaging effect of the stress of immobilization and the ischemia of tissues and cells was estimated by activity of cytolytic enzymes in the blood plasma and by the concentration of molecules of average and low molecular mass in the blood plasma and erythrocytes. The activity of enzymes of an aspartate aminotransferase (2.6.1.1. AST), alaninaminotranspherases (2.6.1.2. ALT), lactate dehydrogenase (1.1.1.27. LDG) were estimated with the standard sets of reactants of the Vital Diagnostics company (St.Petersburg), the quantity of molecules of average mass (MAM) in the blood plasma and erythrocytes was counted according to the method of M.J.Malahova (15). The optical density was measured on a spectrophotometer of CF-56 LOMO-SPECTRUM.

### The statistical processing of results of the research

The calculations and the statistical processing of results of the research were executed by means of software package StatSoft Statistica 6.0. The average arithmetic value in groups and errors of the arithmetic averages were estimated. The results were analyzed with the use of the one-factorial variance analysis, differences between groups were considered authentic at D<0.05.

## RESULTS AND DISCUSSION

The ischemia arising on the basis of circulation disorder in a shin muscle, owing to the cutting of a femoral artery, is accompanied by an exit in blood of two indicators of cytolysis – LDG and AST, since the activity of these enzymes in the group 3 is authentically higher, than with the intact animals. The stress influence caused by a short-term immobilization of an animal on a laboratory table, causes growth of activity of enzyme LDG in blood. However, with an immobilization, unlike with ischemia, the discharge of LDG is considered to be the most likely not from a shin muscle, but from a myocardium as more subject to destructive changes at stress, than skeletal muscles. The influence of a field of electric impulses after the modeling of an ischemia of a shin muscle does not aggravate the damage caused by ischemia since the activity of AST, ALT and LDG in the group 4 authentically does not differ from the same indicators of the group 3 (Table 1).

**Table 1:** Biochemical indicators of blood of experimental animals

Indicator		Groups			
		1	2	3	4

LDG, U/l		165.2 ± 34.6	280.2 ± 14.0	276.1 ± 7.1	309.5 ± 35.5
AST, U/l		12.7 ± 1.7	11.5 ± 0.7	17.4 ± 0.8	17.1 ± 0.8
ALT, U/l		10.5 ± 0.7	13.4 ± 0.1	10.1 ± 0.9	12.6 ± 2.0
MAM	254 nm	0.159 ± 0.003	0.132 ± 0.006	0.077 ± 0.008	0.140 ± 0.009
In plasma, standard unit.	280 nm	0.137 ± 0.004	0.178 ± 0.002	0.115 ± 0.007	0.202 ± 0.009
MAM	254 nm	0.702 ± 0.009	0.685 ± 0.011	0.684 ± 0.028	0.566 ± 0.033
In erythrocytes, standard unit.	280 nm	0.247 ± 0.003	0.253 ± 0.008	0.291 ± 0.011	0.251 ± 0.008

It is known that the hypoxia of tissue developing because of the ischemic damage, the integrity disorder of membranes of cells, their destruction promote the accumulation in blood underoxidated products of molecules of average and low molecular mass (MAM), including both products of disintegration of cells, and many factors of inflammation, proinflammatory cytokines. In the Table 1 there is the data about the change of MAM – endogenous toxic products (regulatory, protective substances, products of the destruction of cells) which are allocated into blood because of various damaging factors and can settle on membranes of cells, accelerating their cytolysis (15). MAM absorb substances of peptide nature with the wavelength of 254 nanometers, and with the wavelength of 280 nanometers – combinations with aromatic rings, including noradrenalin and adrenaline production of which amplifies with the activation of sympathetic department for the purpose of nonspecific adaptation to the stress factors action.

From the analysis of the data in the Table 1, it follows that the ischemic damage of a shin (group 3) is accompanied by sedimentation on membranes of erythrocytes of MAM absorbing at 280 nanometers, and immobilization (group 2) is accompanied by accumulation of similar products in blood plasma in comparison with the group of intact rats. After the influence by a field of electric impulses on the animals with a shin ischemia (group 4) the quantity of molecules of average mass on membranes of erythrocytes decreases to the level of these indicators of the intact animals, but the level of MAM in blood plasma stays high, measured at 280 nanometers.

The reason of the hyperactivity of LDG and the level of MAM in blood plasma of the rats treated after the ischemia in comparison with the group of the intact animals might be the 15-minutes-long fivefold immobilization of the animals, necessary for the procedure of influence by the field of electric impulses. The immobilization even without additional influence causes nonspecific activation of the sympathetic department of vegetative nervous system that can be followed by damage of structures of various organs, augmented permeability of membranes of cardiac hystiocytes and accumulation of products of endogenous intoxication, including catecholamines absorbing at 280 nanometers. Hence, the augmented activity of LDG of the treated animals and the level of MAM in their blood plasma are not bound to the influence of the field of electric impulses. On the contrary, influence by the spatially distributed field promotes authentic reduction of MAM on membranes of erythrocytes of the treated rats (group 4) in comparison with not treated animals (group 3).

The data of the biochemical research is confirmed by the histological and immune chemical research of the ischemic muscle preparations.

**Figure 1:**
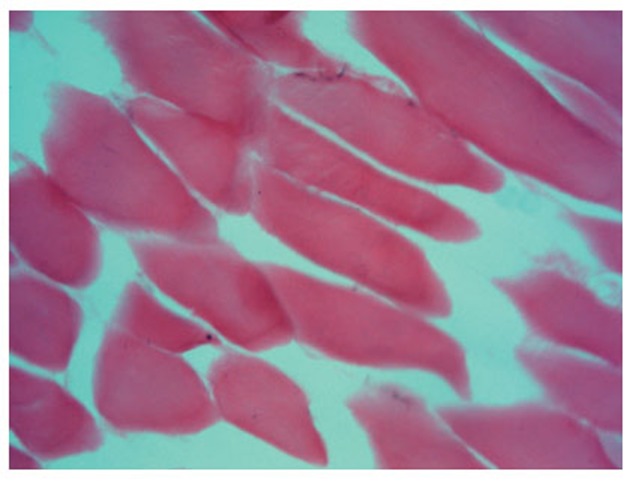
A longitudinal section of the ischemic muscle (×200): the intersticial edema, destruction of kernels and muscular plasmodium.

**Figure 2:**
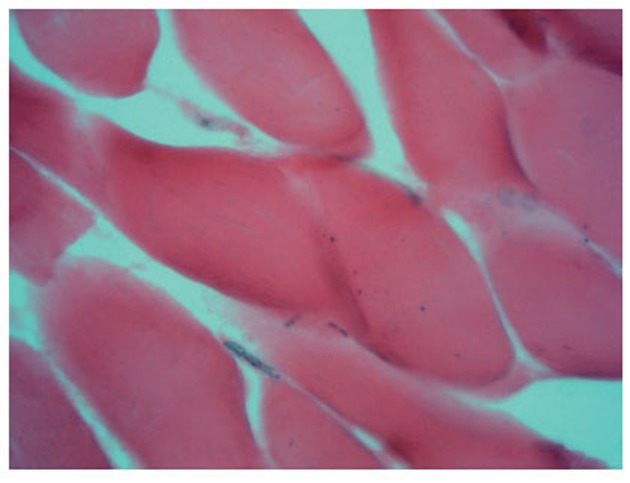
A cross-section of the ischemic muscle (×400): signs of a myomalacia of a muscular fiber in the form of disappearance of cross-section striation, with isolated openings in capillaries in endomysium.

The morphological picture of sections of the ischemic muscles shows clearly the interstitial edema of the skeletal muscular tissue, the structures of endomysum are dilated with isolated openings in capillaries (Fig. 1). The myomalacia with loss of the sarcomere structure (Fig. 2) is observed.

**Figure 3:**
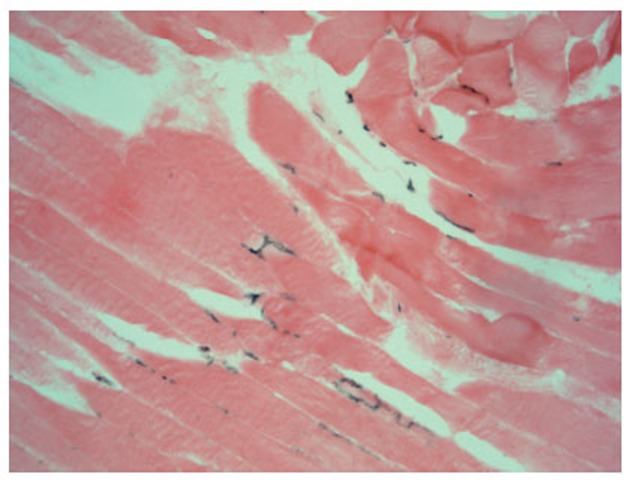
A longitudinal section (enlargement ×200): an ischemic muscle after the influence by the field: kernels of the plasmodium are kept, cross-section striation remains in the majority of fibers with multiple openings in the capillaries in endomysium.

**Figure 4:**
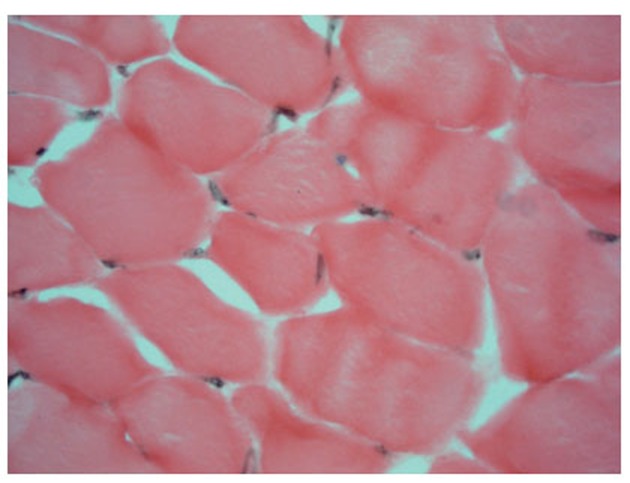
A cross-section (enlargement ×400): an ischemic muscle after the course of FRF.

On the sections of ischemic shin muscles of the rat subjected to influence of the fields it is visible that the edema, characteristic for an ischemic lesion, is reduced, neogenesis signs of the retractor apparatus of muscular fiber in the form of visual restoration of cross-section striation (Fig. 3, 4) are defined. All preparations (Fig. 1–4) were colored on an alkaline phosphatase according to the method of Gomori which allowed to see a larger quantity of endotheliocytes on the preparations 3 and 4 in comparison with the preparations 1 and 2.

The quantity of capillaries was counted at a staining of preparations on CD31 – a specific marker of endotheliocytes. The Table 2 shows that ischemia causes authentic reduction of the quantity of capillaries. It is revealed that in ischemic muscles of the treated animals the quantity of capillaries is authentically higher, than of the intact animals.

**Table 2:** Quantity of capillaries in a femoral muscle of a rat on 1 mm^2^, identified on the expression of CD31

Indicator	Groups			
	1	2	3	4

Quantity of capillaries	138.82 ± 25.98	126.14 ± 7.5	28.7 ± 1.9	69.79 ± 2.7

These morphological observations can be estimated as intensifying of processes of an angiogenesis and restoration of the retractor apparatus of a muscular fiber in an ischemic shin muscle of a rat under the influence of FRF.

The research of the influence by the spatially distributed rotating field of electric impulses in the projection of cervical ganglions of sympathetic system for correction of a chronic pathology – muscle ischemia, have shown that influence by this field based on the method of dynamic correction of activity of a sympathetic nervous system: promotes blood supply restoration of the ischemic muscles through augmentation of quantity of capillaries and reduction of products of an endogenous intoxication.

## CONCLUSION

The results received in the present work show that application of the spatially distributed rotating field of electric impulses during the influence in a projection of cervical ganglions of sympathetic system stimulates angiogenesis of a muscular tissue of a shin of laboratory rats and promotes reduction of products of an endogenous intoxication. It allows to see this technology as perspective for working out of medical techniques of prevention and treatment of organs damaged by ischemia.
